# Clinical Outcomes Following Intra-Articular Administration of Autologous Muscle-Derived Mesenchymal Stem Cells in Horses with Chronic Osteoarthritis: A Prospective Open-Label Study

**DOI:** 10.3390/ani16101523

**Published:** 2026-05-15

**Authors:** Didier Serteyn, Hélène Graide, Justine Ceusters, Maxime Vandersmissen, Alexandra Salciccia, Charlotte Sandersen, Jean-Philippe Lejeune

**Affiliations:** 1 Center for Oxygen Research and Development, Fundamental and Applied Research for Animals and Health, University of Liege, 4000 Liege, Belgium; helene.graide@uliege.be (H.G.); justine.ceusters@revatis.com (J.C.); charlotte.sandersen@uliege.be (C.S.); 2 Equine Research Center, 6698 Vielsalm, Belgium; jph.lejeune@montlesoie.be; 3 Revatis S.A., 6900 Marche-En-Famenne, Belgium; 4 Equine Clinic, Faculty of Veterinary Medicine, University of Liege, 4000 Liege, Belgium; m.vandersmissen@uliege.be (M.V.); alexandra.salciccia@uliege.be (A.S.)

**Keywords:** osteoarthritis, horse, orthobiologics, mesenchymal stem cells, clinical studies

## Abstract

Mesenchymal stromal/stem cells (MSCs) are increasingly investigated as intra-articular therapies for equine osteoarthritis (OA), although most studies have focused on allogeneic or combination-based approaches. Evidence supporting the use of autologous MSCs as a stand-alone treatment remains limited. The present study evaluated the safety and clinical evolution following intra-articular administration of autologous muscle-derived MSCs (mdMSCs) in horses with naturally occurring chronic OA. Thirteen horses with confirmed clinical disease were included. Each affected joint received a single injection, with the administered cell dose adapted to joint size (1 × 10^7^ or 2 × 10^7^ cells). Clinical assessments were conducted at baseline and at 6 and 12 weeks post-treatment using the American Association of Equine Practitioners (AAEP) lameness scale, together with a joint inflammation score and a composite total clinical score (TCS). Clinical scores decreased over time, with statistically significant improvements observed at both follow-up time points. Seven of thirteen horses met the predefined responder criteria based on AAEP improvement, including complete resolution of lameness in several cases. The treatment was well tolerated, with only mild and transient local reactions that resolved without intervention. These results indicate that intra-articular administration of autologous mdMSCs is associated with clinically relevant improvement in horses with chronic OA.

## Simple Summary

Osteoarthritis is a frequent cause of persistent lameness in horses, and currently available treatments often provide only partial or short-term improvement. In this prospective clinical study, horses affected by chronic osteoarthritis were treated with intra-articular injections of autologous muscle-derived mesenchymal stem cells (mdMSCs) used as a stand-alone therapy. Clinical parameters, including lameness and joint inflammation, were monitored over a 12-week period and showed progressive improvement. The treatment was well tolerated, with no major systemic adverse events reported. These findings support the safety profile and potential clinical benefit of autologous mdMSCs in equine osteoarthritis.

## 1. Introduction

Osteoarthritis (OA) is a major cause of chronic lameness and reduced athletic performance in horses, leading to significant welfare and economic implications [1]. Current therapeutic strategies, including non-steroidal anti-inflammatory drugs and intra-articular corticosteroids, primarily provide symptomatic relief and do not modify the underlying degenerative process [2].

Mesenchymal stromal/stem cells (MSCs) have emerged as a promising biological approach for OA. They act predominantly through immunomodulatory and paracrine mechanisms rather than direct cartilage regeneration. Intra-articular MSC administration is therefore expected to reduce synovial inflammation, alleviate pain, and improve joint function in naturally occurring OA, rather than induce rapid structural cartilage repair [3,4,5].

Recent systematic reviews and meta-analyses of randomized trials in human osteoarthritis support a favorable clinical signal for intra-articular MSC therapy while also highlighting substantial heterogeneity in cell source, dosing, and study design [6]. In equine osteoarthritis, the current evidence base for commonly used intra-articular therapies remains heterogeneous and generally of a low level, although studies of intra-articular MSCs have generally reported clinically favorable outcomes [7].

In horses, a randomized controlled trial has reported improvements in clinical signs following intra-articular administration of an allogenic MSC-based product in combination with plasma-derived components [8]. Systematic reviews confirm an overall beneficial effect on lameness while also highlighting substantial heterogeneity in cell source, dose, manufacturing procedures and outcome measures across studies. Notably, most controlled equine trials have evaluated relatively low cell doses or combination products, limiting the ability to attribute observed effects directly to MSCs alone [9].

Skeletal muscle has been proposed as an attractive source of MSCs for autologous applications, as it allows for minimally invasive harvesting and robust ex vivo expansion. Ceusters et al. (2017) described a reproducible microbiopsy-based method to isolate and expand muscle-derived MSCs (mdMSCs) across species, including the horse, and demonstrated their clonogenicity, trilineage differentiation and typical MSC immunophenotype [10]. More recently, Dechêne et al. (2023) showed that equine mdMSCs exposed in vitro to osteoarthritic cartilage explants secrete proteins with anti-inflammatory, chondroprotective and matrix-modulating properties, providing a mechanistic rationale for their use in OA [11].

Beyond veterinary medicine, naturally occurring equine OA represents a highly relevant large-animal model for translational research, given its anatomical, biomechanical and pathophysiological similarities to human joint disease and the possibility to assess interventions under physiological loading conditions [12]. While intra-articular MSC therapies have shown promising results in human knee OA, heterogeneity across clinical trials has so far limited broad clinical recommendations [13,14,15]. Well-characterized veterinary studies may therefore contribute valuable translational evidence bridging experimental mechanisms and clinical application.

The present prospective clinical trial was designed and conducted in compliance with VICH GL9 Good Clinical Practice guidelines for veterinary clinical studies. It evaluates the safety and clinical effects of a pure autologous mdMSC preparation administered intra-articularly in horses with chronic osteoarthritis, using 1 × 10^7^ cells per administration unit and protocol-adapted dosing according to joint size. Preliminary results from this study were previously presented in abstract form at a scientific meeting; the present manuscript reports the complete clinical dataset and provides a fully developed and original analysis [16].

## 2. Materials and Methods

### 2.1. Study Design and Animals

This study was designed as a prospective, open-label, single-arm exploratory clinical trial in horses with naturally occurring osteoarthritis, conducted in accordance with Good Clinical Practice guidelines. Each horse was followed longitudinally and served as its own control. The study was approved by the competent authorities responsible for animal welfare and veterinary medicinal product regulation. Five veterinary investigators with recognized expertise in equine orthopedics participated in the trial.

Horses were eligible for inclusion (Tinc) based on the presence of osteoarthritis-related lameness persisting for more than 3 months. No intra-articular treatment had been administered within the 3 months preceding inclusion.

Following inclusion, a manufacturing period of approximately 6 to 8 weeks was required before treatment. During this interval, no intra-articular or systemic treatments likely to influence clinical outcomes were permitted.

Sixteen client-owned horses were enrolled after informed owner consent was obtained. Of these, 13 completed the study and were included in the final efficacy analysis and are presented in Table 1.

Treatment (T0) corresponded to the day of intra-articular mdMSC administration. Clinical status was subsequently evaluated longitudinally within each horse.

From inclusion (Tinc) through follow-up, horses were maintained under controlled exercise conditions (restricted activity or light work), as determined by the attending clinician to minimize variability related to workload. No systemic or intra-articular anti-inflammatory treatments were permitted during this period.

### 2.2. Muscle Microbiopsy and Cell Culture

At the inclusion time (Tinc), a muscle sample was collected using a minimally invasive microbiopsy technique under light sedation with detomidine (10 µg/kg IV) and local anesthesia (1 mL mepivacaine, subcutaneous). A small fragment of tissue (approximately 20 mg) was obtained from the triceps brachii and immediately placed in transport medium for further processing [10]. The interval between inclusion (Tinc) and treatment (T0) corresponded to the cell manufacturing period and lasted approximately 6 to 8 weeks, with limited variability between cases. Adherent cells were obtained and subsequently expanded under GMP-compliant conditions. After this expansion phase, a total of 4–6 × 10^7^ autologous mdMSCs were available for clinical use [10]. The final cell product (drug product) was evaluated for appearance, identity, purity, microbiological safety, viability, and cell count. Visual inspection was performed to confirm the absence of turbidity and the presence of adherent spindle-shaped cells. Immunophenotypic characterization was assessed by flow cytometry (FACS), confirming expression of mesenchymal markers (CD90, CD44 ≥ 70%) and low expression of hematopoietic markers (CD45, HLA-DR ≤ 2%). Microbiological quality control included sterility testing (BacT/ALERT^®^, European Pharmacopoeia 2.6.27), mycoplasma detection (PCR-ELISA, European Pharmacopoeia 2.6.7), and endotoxin quantification (LAL assay, European Pharmacopoeia 2.6.14), all required to be within acceptable limits. Cell viability was assessed using Trypan Blue exclusion (European Pharmacopoeia 2.7.29), with a predefined acceptance threshold of >75%. Cell count was determined prior to administration. Only batches meeting all predefined release specifications were approved for clinical use [10,17]. All release criteria were evaluated against predefined specifications prior to administration.

### 2.3. Product, Dose and Administration

On the day of treatment (T0), a single intra-articular injection of autologous mdMSCs was performed per affected joint using cryovials each containing 1 × 10^7^ viable autologous mdMSCs, thawed, washed, and resuspended in 1 mL of CryoStor CS5^®,^ (Biolife Solution, Inc, Bothel, WA, USA). The administered dose was adapted to joint size: 1 × 10^7^ cells for metacarpophalangeal/metatarsophalangeal (fetlock) and distal interphalangeal joints and 2 × 10^7^ cells for stifle joints.

This study was not designed to evaluate dose–response effects. No concomitant intra-articular medication was permitted.

### 2.4. Clinical Evaluation

Clinical assessments were carried out by experienced equine clinicians at each participating center using standardized scoring systems. Lameness was evaluated using the American Association of Equine Practitioners (AAEP) scale (0–5).

Joint pain and swelling were assessed by palpation and manipulation using a semi-quantitative score ranging from 0 (none) to 3 (severe). The total clinical score (TCS; range 0–8) corresponded to the sum of the AAEP score and the inflammation score (IS).

Clinical evaluations were performed at inclusion (Tinc), at baseline before injection (T0), and at approximately 6 weeks (T1) and 12 weeks (T2). Individual baseline characteristics, including treated joints, administered dose, and AAEP score at T0, are presented in Table 1.

### 2.5. Endpoints and Statistical Analysis

The primary efficacy assessment focused on clinically meaningful improvement in lameness, as measured by changes in the AAEP score over time. For descriptive clinical interpretation, horses were also classified as responders when they showed a marked improvement in AAEP score of 2 units and/or complete resolution of lameness at the final follow-up.

Because clinical scores were ordinal and repeated measures were obtained from the same horses at T0, T1, and T2, analyses were performed using non-parametric methods. Overall changes across post-treatment time points (T0, T1 and T2) were assessed using the Friedman test for repeated measures. When significant, paired post hoc comparisons between T0 and follow-up visits (T1 and T2) were performed using two-sided Wilcoxon signed-rank tests. The significance threshold was set at α = 0.05. Data are presented as median values with interquartile ranges (IQR). Statistical analyses were performed using Python version 3.11 and SciPy version 1.11.4. AI-assisted tools were used for statistical coding support and language editing; all analyses and interpretations were performed and validated by the authors.

## 3. Results

Thirteen horses completed follow-up through week 12 (T2). Three horses were retired by their owners before the first injection. All administered mdMSC batches met predefined release criteria. Visual inspection confirmed appropriate cell morphology and absence of turbidity in all preparations. Immunophenotypic analysis showed consistent expression of mesenchymal markers (CD90 and CD44) above acceptance thresholds, with minimal expression of hematopoietic markers (CD45 and HLA-DR). Sterility and mycoplasma testing were negative for all batches, and endotoxin levels were below the accepted limits. Post-thaw viability exceeded the predefined threshold in all cases (>75%).

All intra-articular injections were performed without technical difficulty. Mild to moderate transient joint swelling was observed in three horses following intra-articular injection. These local reactions resolved spontaneously within a few days without any specific treatment. Mild, transient joint effusion was observed exclusively in fetlock joints in three horses. This consisted of a slight distension of the synovial recesses detectable the day following injection, without any associated worsening of lameness. In all cases, the reaction resolved spontaneously within 48 h without treatment. No specific safety signal was observed in joints receiving two administration units.

Seven of thirteen horses met the predefined composite responder criteria, defined as either complete resolution of lameness (AAEP score = 0 at T2; *n* = 5) or a reduction of at least two AAEP grades (*n* = 2). Among the remaining horses, four showed stable lameness scores throughout follow-up, while two exhibited a moderate improvement corresponding to a one-grade reduction in AAEP score, without reaching the responder threshold.

Clinical outcome data are summarized in Table 2 and presented as median values with interquartile ranges. Overall changes across post-treatment time points (T0, T1, T2) were assessed using the Friedman test for repeated measures. A statistically significant effect of time was observed for all three parameters (AAEP, IS and TCS). Post hoc paired comparisons using Wilcoxon signed-rank tests (two-sided) showed significant improvements at both 6 weeks (T1) and 12 weeks (T2) compared with baseline at injection (T0).

**Figure 1:**
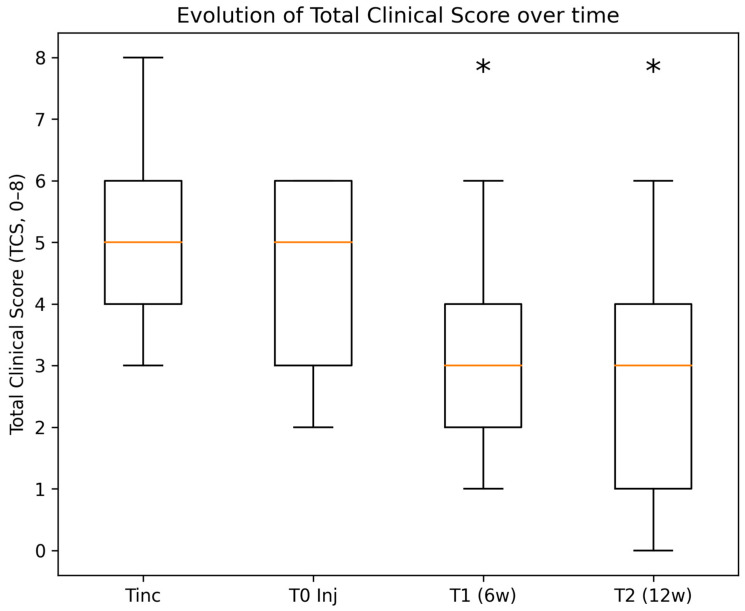
Evolution of the Total Clinical Score (TCS = AAEP + IS; range 0–8) from inclusion (Tinc) to baseline at injection (T0 Inj), and at 6 weeks (T1) and 12 weeks (T2) after intra-articular mdMSC administration. Boxplots represent median values (horizontal lines), interquartile ranges (boxes), and ranges (whiskers). * indicates a statistically significant difference compared with T0 Inj (Wilcoxon signed-rank test, two-sided, *p* < 0.05).

All horses had a documented history of osteoarthritis-related lameness for more than three months prior to inclusion, followed by an additional manufacturing period of approximately two months before intra-articular mdMSC injection. After treatment, a consistent reduction in clinical scores was observed at both 6 and 12 weeks post-injection. Figure 1 illustrates the evolution of the Total Clinical Score (TCS) over time.

## 4. Discussion

The results of the present study show that intra-articular administration of autologous mdMSCs was associated with clinically meaningful improvement in horses with chronic osteoarthritis, with more than half of the horses meeting a stringent composite responder definition and significant reductions in lameness, joint inflammation, and total clinical score at both 6 and 12 weeks. These outcomes are notable given the chronicity of disease, the inclusion of client-owned horses under real-world conditions, and the absence of concomitant intra-articular medications, which reduces confounding by other orthobiologic agents. Recent human meta-analytic evidence focusing specifically on intra-articular MSC injections administered without concomitant therapeutic confounders further supports the clinical relevance of assessing MSCs as a stand-alone intervention [18].

The inclusion of horses with documented osteoarthritis-related lameness for more than three months, followed by a manufacturing period before treatment, supports the chronic nature of the study population. The study was not designed to formally assess spontaneous disease evolution before treatment. However, clinical improvement was observed following intra-articular mdMSC administration.

Previous equine studies have reported beneficial effects of intra-articular MSC-based therapies for osteoarthritis, primarily using allogeneic cell sources and frequently combining cells with plasma-derived products [8,19]. While these approaches have shown promise, heterogeneity in cell source, dose, manufacturing protocols and outcome measures has complicated cross-study comparisons and limited attribution of efficacy to the cells themselves [7]. In this context, the present study extends the existing literature by providing prospective clinical data for a pure autologous mdMSC product, administered at a cell dose higher than that commonly reported in previous controlled equine trials. This design reduces confounding by concomitant orthobiologic agents and supports attribution of the observed clinical signal to the administered cells.

The choice of skeletal muscle as a cell source and the use of a minimally invasive microbiopsy protocol are supported by sufficient methodological and mechanistic considerations to justify this prospective clinical study and corroborate the observed results [10,11,19].

Current reviews suggest that the clinical activity of intra-articularly injected MSCs is more likely to depend on paracrine, immunomodulatory, and pain-modulating effects than on direct structural engraftment alone [20]. The temporal pattern of improvement observed in this study is consistent with the current understanding. In osteoarthritis, MSCs are considered to act mainly through paracrine and immunomodulatory pathways rather than through direct cartilage regeneration. MSC-derived mediators contribute to the reduction in synovial inflammation, influence local immune responses within the joint, and limit catabolic activity in resident chondrocytes [5,20]. The sustained improvement after 6 weeks up to 12 weeks in our cohort is therefore consistent with a scenario in which a single intra-articular dose of mdMSCs triggers a cascade of paracrine and immunomodulatory events that dampen inflammation and support cartilage homeostasis, rather than rapidly rebuilding cartilage structure.

From an immunological standpoint, autologous mdMSCs offer several theoretical advantages over allogeneic products. Previous studies have reported that repeated intra-articular administration of allogeneic MSCs is generally well tolerated in horses and is not associated with detectable cellular or humoral immune responses in most cases [21]. However, more recent in vivo findings suggest that the systemic cellular immune response to MSCs may vary depending on the inflammatory status of the recipient, as well as the differentiation or preconditioning state of the cells and their MHC compatibility. In a horse model, allogeneic MSCs induced disTinct lymphocyte activation profiles depending on these parameters, suggesting that certain combinations of high inflammation, advanced differentiation and MHC mismatch may increase immunogenicity [22]. In contrast, the present study employed a pure autologous mdMSC product with a well-defined phenotype in naturally occurring OA, eliminating the risk of allo-immunization and contributing to the favorable safety profile observed.

The equine OA model continues to be highly relevant for translational research because of its anatomical, biomechanical and pathophysiological similarities to human joint disease and because it allows for evaluation under realistic loading conditions. By providing data in a spontaneous large-animal model, this study strengthens the translational bridge between mechanistic insights, veterinary application and future human clinical development of autologous mdMSC therapies.

This study has several strengths, including the prospective evaluation of client-owned horses with chronic, naturally occurring osteoarthritis, the use of a pure autologous muscle-derived MSC product without concomitant intra-articular orthobiologic confounders, a clinically meaningful responder analysis, and an overall favorable safety profile. However, several limitations should also be acknowledged, including the open-label single-arm design, the relatively short follow-up period of 12 weeks, the limited sample size and the heterogeneity of treated joints. Formal inter-observer variability was not evaluated, which represents a limitation of the study. Clinical scoring systems could be complemented in future studies by objective gait analysis.

In conclusion, intra-articular administration of autologous mdMSCs was well tolerated and was associated with statistically significant and clinically meaningful improvement in horses with chronic osteoarthritis. The predefined primary endpoint, based on improvement in AAEP lameness score, was achieved, and more than half of the treated horses met responder criteria, with complete resolution of lameness observed in a subset of cases. Consistent improvements were also observed across secondary clinical parameters, including joint inflammation and total clinical scores. Overall, these findings indicate a potential clinical benefit of autologous mdMSCs when used as a stand-alone intra-articular treatment for equine osteoarthritis.

## Figures and Tables

**Table 1:** Baseline characteristics of the study population, including treated joints, administered cell dose, and initial lameness severity (AAEP score at T0).

Horse	Breed	Sex	Age (Years)	Joint	Dose (×10^7^ mdMSCs)	AAEP Score (T0)
1	Pura Raza Española (PRE)	Gelding	24	Left forelimb fetlock	1	4
2	Belgian Warmblood horse	Gelding	10	Left forelimb proximal interphalangeal joint	1	3
3	Thoroughbred	Gelding	9	Right forelimb fetlock	1	4
4	Lusitano	Mare	7	Left hindlimb fetlock	1	3
5	Luxembourg Warmblood	Gelding	10	Left forelimb fetlock	1	3
6	Belgian Warmblood horse	Stallion	14	Left medial femoro-tibial joint	2	2
7	Belgian Warmblood horse	Gelding	12	Left medial femoro-tibial joint	2	2
8	Thoroughbred	Mare	8	Right medial femoro-tibial joint	2	2
9	French saddle horse	Mare	11	Left forelimb distal interphalangeal joint	1	2
10	Hannover	Gelding	5	Left forelimb fetlock	1	3
11	KWPN	Stallion	12	Right medial femoro-tibial joint	2	2
12	Arabian Thoroughbred	Stallion	10	Left medial femoro-tibial joint	2	3
13	Belgian Warmblood horse	Mare	10	Right medial femoro-tibial joint	2	2

**Table 2:** Data are presented as median [interquartile range]. Overall changes across post-treatment time points (T0, T1, T2) were assessed using the Friedman test for repeated measures. Paired post hoc comparisons were performed using Wilcoxon signed-rank tests (two-sided). Statistical significance was set at *p* < 0.05.

Endpoint	T0 Median [IQR]	T1 Median [IQR]	T2 Median [IQR]	Friedman χ^2^ (T0–T2)	*p* (Friedman)	*p* (Wilcoxon T0–T1)	*p* (Wilcoxon T0–T2)
AAEP	3.0 [2.0–3.0]	2.0 [1.0–3.0]	1.0 [0.0–3.0]	16.71	0.00024	0.0039	0.0067
IS	2.0 [1.0–2.0]	1.0 [1.0–2.0]	1.0 [0.0–2.0]	14.31	0.00078	0.0047	0.0050
TCS	5.0 [3.0–5.0]	3.0 [2.0–4.0]	3.0 [1.0–4.0]	19.00	0.000075	0.0024	0.0031

## Data Availability

The data generated in this study form part of a regulatory submission to the European Medicines Agency (EMA), conducted under the supervision of the Belgian Federal Agency for Medicines and Health Products (FAMHP/AFMPS) (authorization numbers 0007367 and 0008679). Due to regulatory and confidentiality constraints related to the marketing authorization process, these data are not publicly available.
